# Large-scale nanoporous metal-coated silica aerogels for high SERS effect improvement

**DOI:** 10.1038/s41598-018-33539-z

**Published:** 2018-10-11

**Authors:** Changwook Kim, Seunghwa Baek, Yunha Ryu, Yeonhong Kim, Dongheok Shin, Chang-Won Lee, Wounjhang Park, Augustine M. Urbas, Gumin Kang, Kyoungsik Kim

**Affiliations:** 1 School of Mechanical Engineering, Yonsei University, 50 Yonsei-ro, Seodaemun-gu, Seoul 120-749 Republic of Korea; 2 School of Basic Sciences, Hanbat National University, Daejeon, Republic of Korea; 3 Department of Electrical, Computer & Energy Engineering, University of Colorado, Boulder, CO 80309 USA; 4 Materials and Manufacturing Directorate, Air Force Research Laboratory, Wright-Patterson AFB, OH 45433 USA; 5 Nanophotonics Research Center, Korea Institute of Science and Technology (KIST), Seoul, 02792 Republic of Korea

## Abstract

We investigate the optical properties and surface-enhanced Raman scattering (SERS) characteristics of metal-coated silica aerogels. Silica aerogels were fabricated by easily scalable sol-gel and supercritical drying processes. Metallic nanogaps were formed on the top surface of the nanoporous silica network by controlling the thickness of the metal layer. The optimized metallic nanogap structure enabled strong confinement of light inside the gaps, which is a suitable property for SERS effect. We experimentally evaluated the SERS enhancement factor with the use of benzenethiol as a probe molecule. The enhancement factor reached 7.9 × 10^7^ when molecules were adsorbed on the surface of the 30 nm silver-coated aerogel. We also theoretically investigated the electric field distribution dependence on the structural geometry and substrate indices. On the basis of FDTD simulations, we concluded that the electric field was highly amplified in the vicinity of the target analyte owing to a combination of the aerogel’s ultralow refractive index and the high-density metallic nanogaps. The aerogel substrate with metallic nanogaps shows great potential for use as an inexpensive, highly sensitive SERS platform to detect environmental and biological target molecules.

## Introduction

Recently, low-cost, large-area plasmonic sensing platforms have been intensively studied for high-throughput chemical and biological analysis. Although the electric field distribution is considerably changed by the substrate index, the importance of substrate refractive indices has been overlooked in previous studies. For example, the light-matter interaction volume in nanoplasmonic sensing systems is substantially changed by substrate indices. In the refractive index sensing of the surrounding medium and detection of the surface absorbed biological molecules, sensitivities are improved on low index substrates (Teflon, n ~ 1.3) as a consequence of greater pushing of the electric field towards the sensing region compared with that of higher index substrates (TiO_2_, n ~ 2.4)^1,2^. Surface-enhanced Raman scattering (SERS) sensing, based on cubic metal nanoparticles, also benefits from lower index substrates such as glass (n ~ 1.5)^3,4^ and flower-like alumina-coated etched aluminum foil (n ~ 1.4)^5^.

Plasmonic nanostructures used in optical sensing support quite different electromagnetic mode profiles. Plasmonic index sensing platforms generate an anti-symmetric mode across a thin metal film^1^, whereas nanocube-based SERS platforms support symmetric modes to enhance the far-field signal^3,4^. One approach is to use a low-refractive index material as a substrate to increase the electric field intensity at the sensing area.

Numerous studies have been performed to develop low refractive index materials (n = 1.1–1.3) including porous silica, sponge-like block copolymers, glancing angle deposited nanowires^6^. Among low refractive index solid-phase materials, silica aerogels have the lowest refractive index (less than 1.01) and high optical transparency over 90%^7^. Silica aerogels, a porous bulk material made up of nanometer-scale silica chain networks, are also uniquely lightweight and superb thermal insulators. The porosity of these materials exceeds 98.5% and their thermal conductivity is as low as 0.25 Wm^−1^ K^−1^^8–10^. A number of industrial and aerospace applications have been demonstrated, including supercapacitors, fuel storage, catalysts, acoustic impedance matching transducers, cosmic dust collectors, and thermal insulating materials used on the space shuttle^11^. The optical properties of silica aerogels have been extensively studied by absorption spectroscopy^12,13^ and photoluminescence in a powdered form^14^. The behavior of silica aerogels as quantum yield enhancers^15^ has also received attention together with their Raman scattering phenomena^16–22^. However, the use of silica aerogels in optical sensing platforms has not yet been considered despite the unique optical properties of these materials. Here, we apply a metal-coated silica aerogel as a SERS template, to obtain high SERS effect improvement.

**Figure 1:**
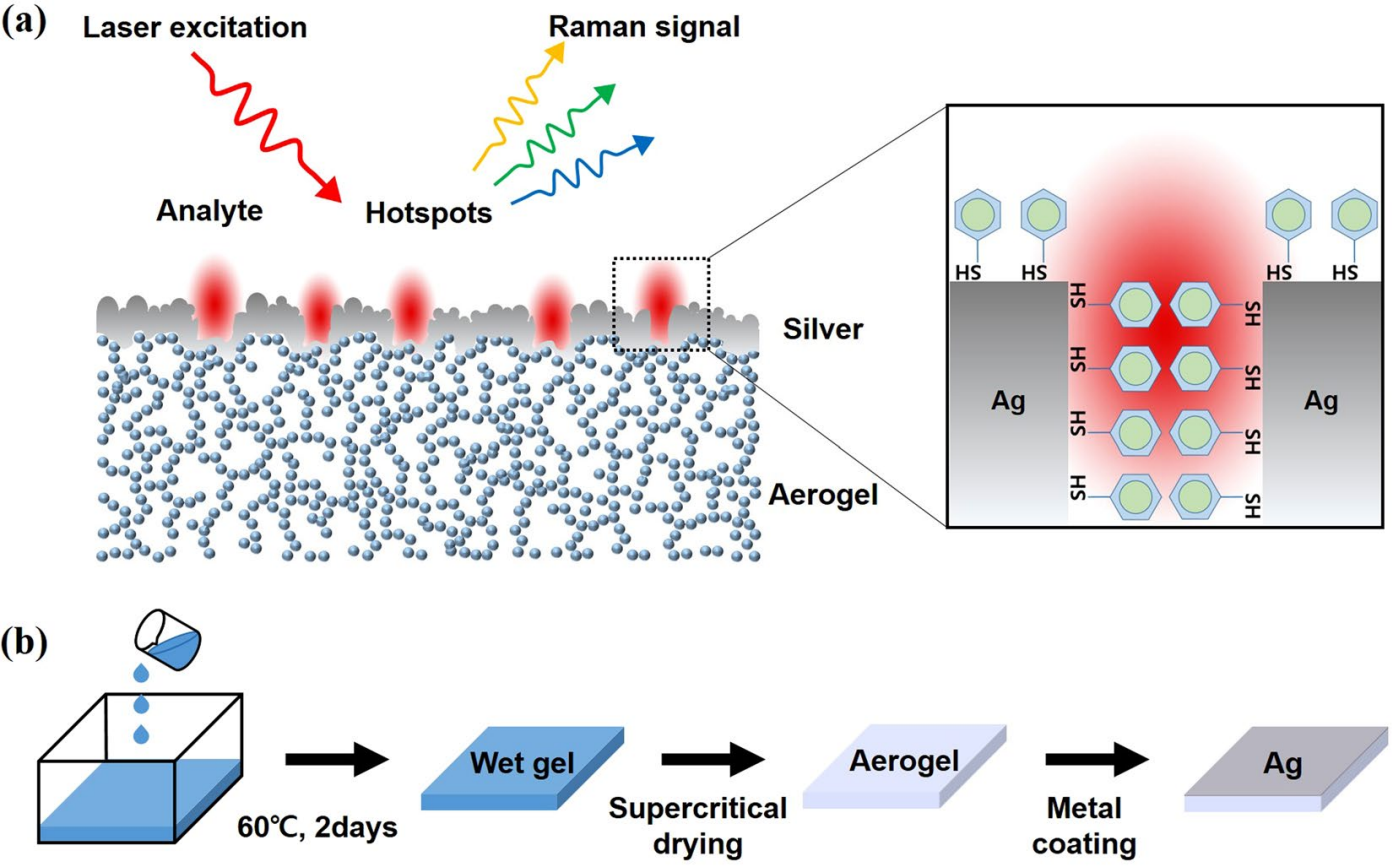
(**a**) Illustration of plasmon-enhanced Raman scattering mechanism for analytes on top of silver-coated silica aerogel. (**b**) Fabrication procedure of silver-coated silica aerogel structure for Raman detection. Silica wet gel was prepared by mixing MTMS, surfactant, and acetic acid. Aerogels were obtained by drying the wet gel with supercritical carbon dioxide at 80 °C, 13.5 MPa. A thin silver film was deposited onto the aerogels by electron beam evaporation to create plasmonic nanogap-based SERS active substrate.

Because the averaged electromagnetic energy density in a material is proportional to the material’s dielectric permittivity and its dispersion^23,24^, the electric field inside the silica aerogel could be smaller than that of other higher refractive index materials. When a silica aerogel is attached to a semi-infinite metal, most of the electric field concentrates at the interface between the aerogel and metal with a long plasmon fringing field depth toward the silica aerogel. If the metal layer is sufficiently thin, for example, thickness ${\rm{t}}\ll {\rm{\lambda }}\,({\rm{wavelength}})$, the electric field is strongly confined at the metal layer with symmetric or anti-symmetric modes of the surface plasmons and a fringing electric field depth stretched both toward the air and silica aerogel. Considering the law of energy conservation, the extruded electromagnetic energy of the silica aerogel transferred to the other side, i.e., the air/metal interface where analyte molecules are located, as described in Fig. 1a. Therefore, if arranged correctly, it is possible to achieve a localized and enhanced electric field at the location of the target molecule.

In this study, we fabricated a high-density metal nanogap structure on ultra-low index substrates by a low-cost, simple, and scalable manufacturing process, for potential use as a SERS template. We theoretically and experimentally studied how the ultralow index and nanoscale roughness of the silica aerogels affected the electric field distribution and improved SERS sensitivity. We found that the SERS enhancement factor for benzenethiol exceeded $7.9\,\times {10}^{7}$ owing to optimization of the density and size of the metallic nanogaps.

## Experimental

### Aerogel and sample fabrication

Figure 1a shows a schematic cross-section of the metal-coated silica aerogel with adsorbed benzenethiol molecules. A schematic diagram of the fabrication process for the silica aerogel and SERS template is shown in Fig. 1b. We placed 0.5 g of urea, 4.76 g of methyltrimethoxysilane (MTMS), 1.1 g of nonionic surfactant poly(ethylene oxide)-blockpoly(propylene oxide)-block-poly(ethylene oxide) triblock copolymer (Pluronic F127) in 7.0 g of 10 mM acetic acid solution^7^. The mixture was stirred at room temperature for 30 min, then slowly poured into a Petri dish. The Petri dish was covered and placed in an oven at 60 °C for 2 days to form a solid gel. The gel was immersed in distilled water for 1 day to eliminate residual chemicals held within the silica chain network. To exchange the solvent with isopropyl alcohol (IPA), the gel was soaked again in isopropyl alcohol (IPA) at 60 °C for 2 days. Subsequently, the gel was maintained in a chamber with liquid carbon dioxide at 80 °C, and 135 bar as the supercritical drying conditions. The average pore size, porosity, and specific surface area of the fabricated silica aerogels were 59.5 nm, 83.9%, 575 m^2^/g, respectively^7^. To prepare SERS-active substrates, 30 or 60 nm of silver (or gold) was deposited onto the aerogel surface by an electron beam evaporator. The two different thicknesses of the metal film were deposited to control the surface structure of the metallic layer on the aerogel substrate.

### Structural and optical characterization and SERS

Structures of bare and metal-coated aerogels were characterized with a scanning electron microscope and focused ion beam (SEM/FIB, Nova 200 NanoLab, FEI Company). We used UV/VIS – NIR Spectrophotometer (UV3600, Shimadzu Scientific Instruments) with an integrating sphere (MPC–3100) to obtain the total reflectance and transmittance spectra. We selected benzenethiols (BZTs) as Raman probe molecules, which were adsorbed onto bare aerogel, metal-coated aerogels, and glass. The BZT molecules formed well-ordered self-assembled monolayers (SAMs) on the gold (silver) surfaces through strong S-Au (S-Ag) bonds both in the vapor phase and in liquid environments^25–29^. Moreover, these molecules can be modified by a various specific functional groups such as 4-MBA or 4-MPBA to promote the binding of target analytes for pH detection or glucose sensing^30,31^. A drop of BZT on a petri dish and the SERS substrates were left in a desiccator. A mechanical pump was used to evacuate the desiccator and produce a BZT-vapor saturated environment. The SERS substrate was left in the desiccator overnight for the BZT to adsorb to the surface of the substrate. Benzenethiol formed a monolayer on the surfaces with a surface density of 0.45 nmol/cm^2 ^^32^. Raman spectra were collected with a Lab Ram ARAMIS Raman spectrometer (Horiba Jobin Yvon). A He-Ne laser (633 nm) was used as a light source for excitation.

## Result and Discussion

### Ultralow refractive index of aerogel

**Figure 2:**
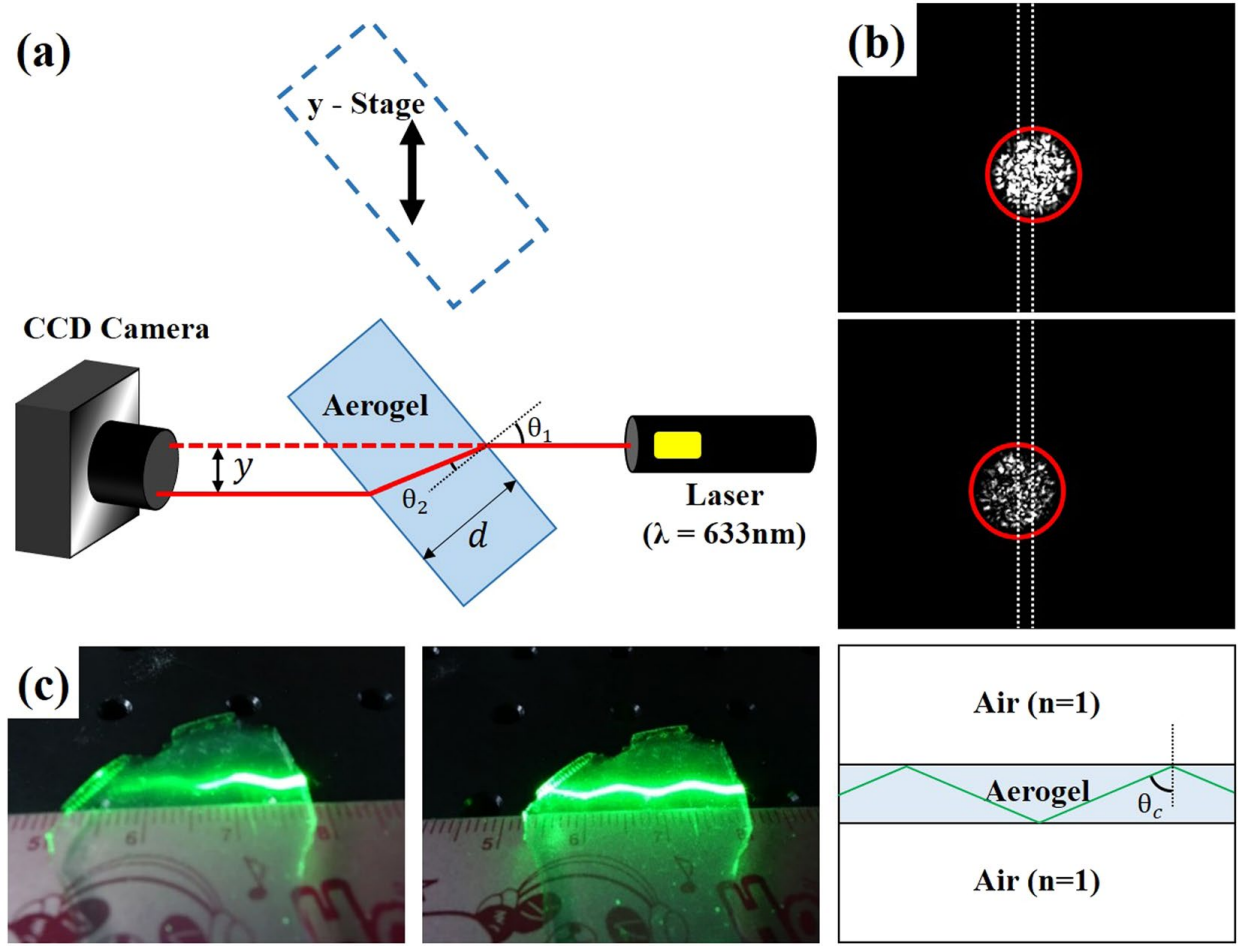
(**a**) Schematic of the experimental setup for measuring the refractive index of the aerogel. Refractive index of the aerogel was calculated by Snell’s law. (**b**) CCD camera image of the shifted laser beam after pass through the aerogel block. (**c**) Picture of non-guided (left) and guided light (right) inside the aerogel via total internal reflection. Refractive index of the aerogel can be calculated from the measured critical angle.

We experimentally measured the refractive index of bulk aerogels with the use of Snell’s law. A schematic diagram is shown in Fig. 2a. A CCD camera was aligned with a He-Ne laser beam (633 nm) propagating in a free-space. We then placed the aerogel sample into the beam path of the laser. The lateral shift (y) of the laser beam, after passing through the aerogel block, with uniform thickness (d), was measured by the CCD camera (see Fig. 2b). We used Snell’s law and trigonometric relationships to determine the aerogel’s refractive index to be 1.08^33^. To confirm the measured index value by another experimental method, the laser beam was coupled from air into the facet of a 1.8 mm-thick aerogel block with a different angle of incidence (see Fig. 2c). When the angle of incidence was greater than 67.1°, total internal reflection occurred at the aerogel/air interface, indicating that the refractive index of the aerogel was 1.08.

### Geometrical structure of aerogel surface

**Figure 3:**
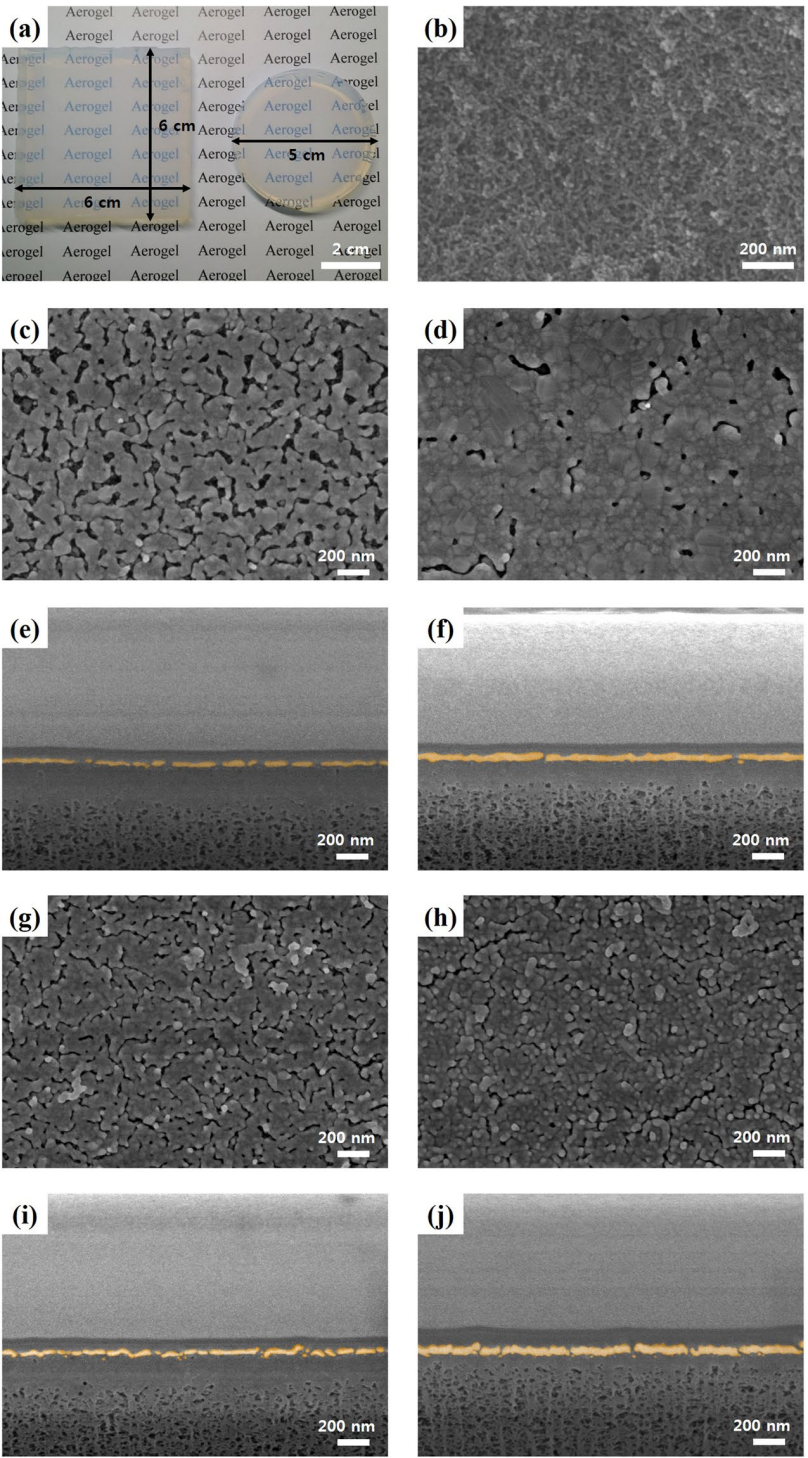
(**a**) Photograph of the fabricated silica aerogel. (**b**) SEM image of the nanoporous aerogel surface. The bright regions represent an area where silica networks are formed. The gray or dark regions represent nanopores. SEM images of silver-coated aerogel surface with different Ag thickness; (**c**) 30, (**d**) 60 nm. False-colored cross-sectional SEM images of (**e**) 30 nm, (**f**) 60 nm silver-coated aerogel. SEM images of gold-coated aerogel surface with different Au thickness; (**g**) 30, (**h**) 60 nm. False-colored cross-sectional SEM images of (**i**) 30 nm, (**j**) 60 nm gold-coated aerogels.

Figure 3a shows a digital camera image of two large silica aerogels. The as-prepared aerogel had a highly mesoporous structure owing to the cross-linked silica chain network, as shown in the SEM image of Fig. 3b^7^. To make the SERS-active substrate, a thin film of silver (Fig. 3c–f) or gold (Fig. 3g–j) was coated on top of the aerogel surface. After metal coating, narrow nanogaps were formed in the metallic thin film. (see Fig. 3c,d,g,h). These metallic nanogaps could strongly concentrate optical fields and thus behaved as Raman-active hot spots, as illustrated in Fig. 1a. The surface morphology of the metallic thin film varied depending on the thickness of the deposited metal. The 30 nm metal-coated aerogels (Fig. 3e,i) exhibited a higher gap density than that of the 60 nm metal-coated aerogels (Fig. 3f,j). Also, 30 nm thick metal-coated aerogels showed higher surface roughness than 60 nm metal-coated aerogels. AFM images with root mean squared roughness (R_q_) are shown in Supplementary Fig. S3. In contrast, the metal layers deposited on glass substrates are planar surface without nanogaps (Supplementary Fig. S1).

And we obtained the cross-sectional SEM images by FIB-SEM system, as shown in Fig. 3e,f,i,j. The images clearly show the thin metal film (false-colored with yellow), nanogaps, and silica chain networks. To avoid the damage of thin metal film morphology on the aerogel during the FIB/SEM measurement, we carried out both E-beam and Ion beam assisted Pt deposition in sequence. The E-beam deposited Pt atoms penetrate into the nanopores of aerogel below the metal film, resulting in the disappearance of porosity of the aerogel substrate. However, from the Ion beam assisted Pt deposition image without E-beam assisted method (see Supplementary Fig. S2), we can clearly see the nanopores of aerogel below the thin metal film.

### FDTD simulation

We used 3D finite-difference time-domain (FDTD) methods to simulate the optical properties of the metal-coated aerogel structures. To investigate the effects of the substrate index on Raman enhancement, we calculated the electric field distributions depending on the substrate index without changing the metallic nanogap structure: aerogel (n = 1.08), glass (n = 1.52), and silicon (n = 3.88)^1,4,34^. In reality, the metal layer deposited on glass or silicon substrate is planar thin film without nanogaps, which are formed only on nanoporous aerogel substrate. For SERS effect, to investigate the effect of lower refractive index substrate, while excluding the different metal layer’s morphology effect, we assumed the virtual cases that the exactly same morphology of metallic nanogap structure is deposited on the substrates with varying refractive index, such as aerogel, glass, silicon. The calculation was performed with commercial FDTD software (Lumerical Inc., Canada).

**Figure 4:**
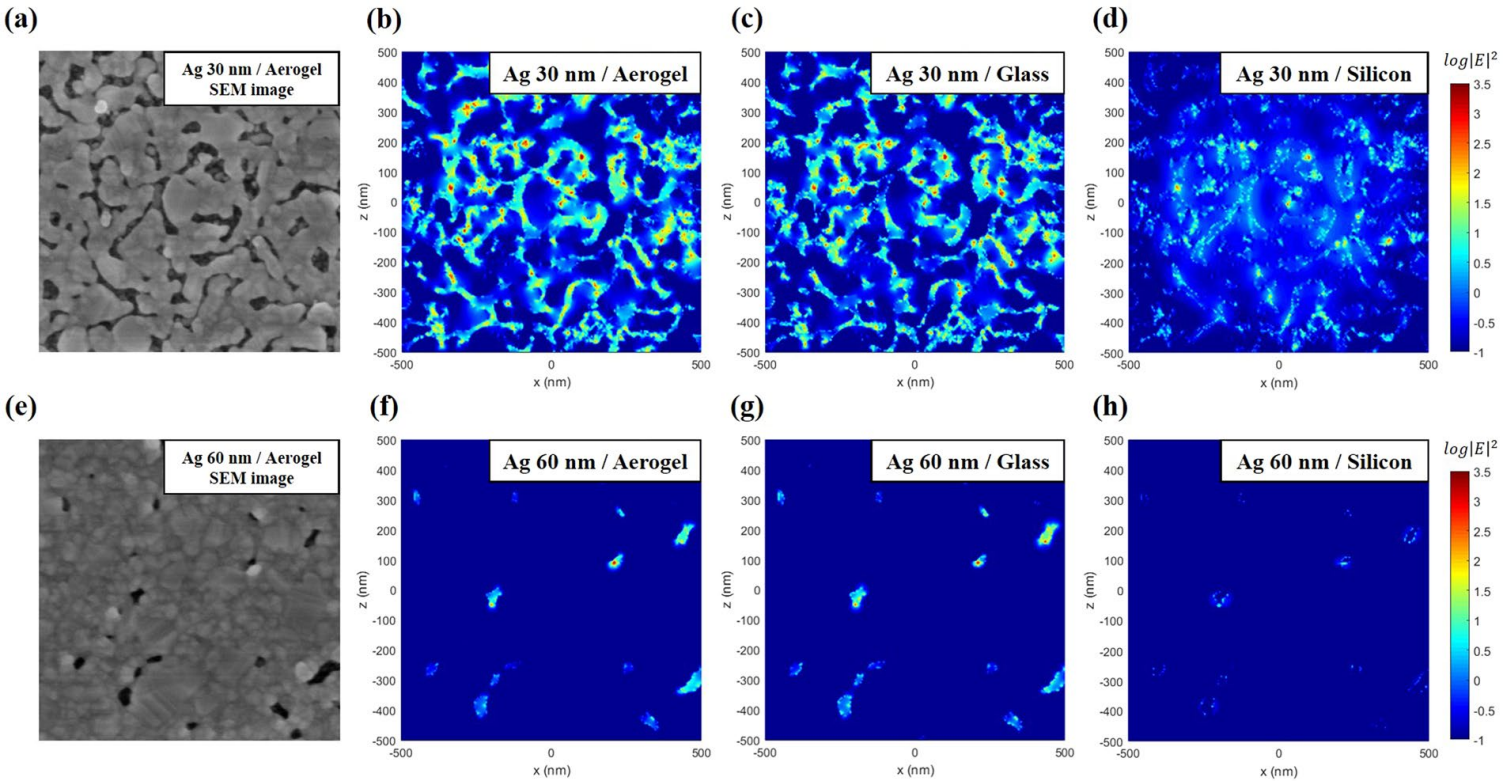
(**a**) SEM image of the 30 nm silver-coated aerogel surface for FDTD simulation. This SEM image was imported into the FDTD simulation. (**b–d**) Electric field profiles by 633 nm light excitation on the top surface of 30 nm silver-coated (**b**) Ag/Aerogel, (**c**) Ag/glass, and (**d**) Ag/Si. (**e**) SEM image of the 60 nm silver-coated aerogel surface for FDTD simulation. (**f**–**h**) Electric field profiles by 633 nm light excitation on the top surface of the 60 nm silver-coated (**f**) Ag/Aerogel, (**g**) Ag/glass, and (**h**) Ag/Si.

We used the top-view SEM images of the metal-coated aerogel to create a model structure (Fig. 4a,e). The images were converted into binary images and then imported into the FDTD software^35^. Figure 4b–d,f,g,h) represent the calculated electric field profile of the 30 nm (60 nm) Ag-coated aerogels, glass, and silicon, respectively, when the 633 nm plane wave was illuminated at normal incidence. For the light excitations by 532 nm and 785 nm plane waves, the calculated electric field profiles are shown in Figs S4, S5 of Supplementary Information, respectively.

For the 30 nm silver nanogap structure on the aerogel substrate illuminated by 633 nm, the maximum field strength in the nanogap compared with that of the incident light is $\frac{\,{|{\rm{E}}|}_{Ag/aerogel}}{{|{\rm{E}}|}_{incident}}=60.3$. Even if we assume that the 30 nm thick silver nanogap structure formed by aerogel is virtually on top of planar glass or silicon substrate with the same morphology, the calculated maximum field strengths are still smaller, i.e., $\frac{\,{|{\rm{E}}|}_{Ag/glass}}{{|{\rm{E}}|}_{incident}}=41.1$ and $\frac{\,{|{\rm{E}}|}_{Ag/Si}}{{|{\rm{E}}|}_{incident}}=25.1$ respectively. These results show that, for the field enhancement, the metallic nanogap geometry formed by aerogel substrate is the main factor; however, the ultralow index substrate also makes a part of contribution.

The SERS enhancement can be approximated by:

$${\rm{EF}}\cong |{\rm{E}}({\rm{\omega }}){|}^{2}|{\rm{E}}(\omega ^{\prime} ){|}^{2},$$

where $E={E}_{loc}/{E}_{inc}({E}_{Loc}$ : local electric field amplitude at the molecule position, ${E}_{inc}$: incident field amplitude), ${\rm{\omega }}$ is the incident lights frequency, and $\omega ^{\prime}$ is the Stokes-shifted frequency. With the assumption that $|{\rm{E}}({\rm{\omega }})|\cong |{\rm{E}}(\omega ^{\prime} )|,\,\,$the enhancement factor follows the so-called, ${\rm{EF}} \sim |{\rm{E}}{|}^{4}$ law^36–39^. Recently developed molecular cavity optomechanics provides an understanding of the strongly coupled vibrational modes of nuclei (phonon) and (cavity) surface plasmon, predicting similar $|{\rm{E}}{|}^{4}$-law-like Raman enhancement under certain conditions^40,41^.

The theoretically estimated SERS enhancement for 30 nm Ag-coated aerogel with 633 nm illumination is ${(\frac{{|{\rm{E}}|}_{Ag/aerogel}}{{|{\rm{E}}|}_{incident}})}^{4}=1.3\times {10}^{7}$, at the air/Ag interface. If we compare the effects of the refractive index between the aerogel and Si substrate, the ratio of the SERS enhancement factor is ${(\frac{{|{\rm{E}}|}_{Ag/aerogel}}{{|{\rm{E}}|}_{Ag/Si}})}^{4}=33.4$. Under 633 nm illumination, both the nanoporous structure and lower refractive index of the aerogel substrate provide higher theoretical SERS EF value. The theoretical SERS EF values of 30 nm Ag-coated aerogel under 532 nm and 785 nm illuminations are obtained as ${(\frac{{|{\rm{E}}|}_{Ag/aerogel}}{{|{\rm{E}}|}_{incident}})}^{4}=2.2\times {10}^{6},\,9.0\times {10}^{7}$, respectively. If we compare the effects of the refractive index between the aerogel and Si substrate, the ratio of the SERS enhancement factor is ${(\frac{{|{\rm{E}}|}_{Ag/aerogel}}{{|{\rm{E}}|}_{Ag/Si}})}^{4}=25.4,\,8.0\times {10}^{3}\,\,$for 532 nm, 785 nm illuminations, respectively. These results show that the use of an aerogel substrate with an ultralow refractive index provides considerable benefits for SERS sensing for broadband light excitations of 532 nm, 633 nm, 785 nm.

### Optical properties

**Figure 5:**
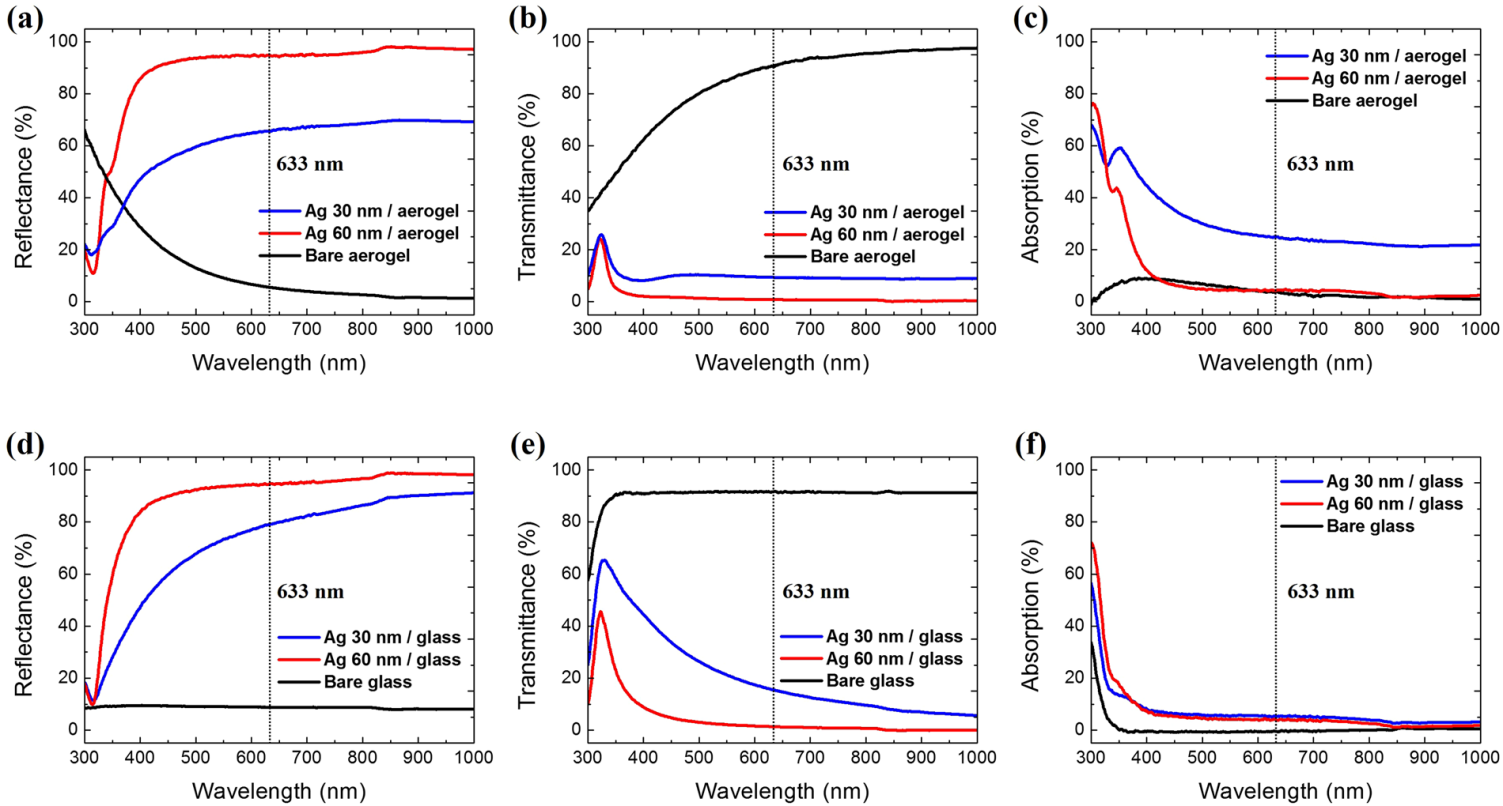
Measured optical (**a**) reflectance, and (**b**) transmittance spectra of the bare aerogel (black), 30 nm silver-coated aerogel (red), and 60 nm silver-coated aerogel (blue). (**c**) Absorption spectra of the bare (black), 30 nm (blue) and 60 nm (red) silver-coated aerogel calculated from 1-R-T. Measured optical (**d**) reflectance, and (**e**) transmittance spectra of the bare glass (black), 30 nm silver-coated glass (red), and 60 nm silver-coated glass (blue). (**f**) Absorption spectra of the bare (black), 30 nm (blue) and 60 nm (red) silver-coated glass calculated from 1-R-T. Black dotted line indicates excitation wavelength (633 nm).

We characterized the optical properties of the bare and metal-coated aerogel in the wavelength range between 300 and 1000 nm. Because the incident light was scattered when it passed through the aerogel, we used a UV-VIS-NIR spectrophotometer fitted with an integrating sphere to measure the total (diffuse + specular) reflectance and transmittance. Because of the ultralow refractive index of the aerogel, which was close to that of air, the Fresnel reflection at the air-aerogel interface was considerably suppressed. As shown in Fig. 5a,b, the bare aerogel exhibited very low reflectance and high transmittance in the visible region. Conversely, the silver-coated aerogels showed increased reflectance and reduced transmittance as the coating thickness increased.

Figure 5c,f show the absorption spectra of the aerogel and silver-coated aerogels obtained from 100–T–R (%). The absorption of the 30 nm silver-coated aerogel was enhanced over a broad wavelength range by the randomly distributed metallic nanogaps^42,43^. The nanogaps generated randomly shaped hot spots with a high density where the electromagnetic field was strongly confined by localized surface plasmon resonance (LSPR)^44^. Interestingly, the aerogel with the 30 nm thick silver coating showed stronger absorption than that of the aerogels with the 60 nm thick silver coating. We attribute this strong absorption to the formation of more plasmonic nanogaps on the surface of the 30 nm silver-coated aerogel compared with the 60 nm silver-coated substrate, as shown in the top-view SEM images (Fig. 3c,d). However, the 60 nm thick Ag-coated aerogel showed similar absorption with bare aerogel in visible wavelength because of the lack of metal nanogaps. The absorption at λ < 400 nm was mainly induced from the interband transition of Ag, not from the LSPR^45^.

For reference, we also measured the optical properties of the 30- and 60 nm-thick silver coatings on planar glass, as shown in Fig. 5d–f. In these cases, no absorption enhancement was observed from hot spots at nanogaps. Similar to silver-coated aerogels, the absorption of the 30 nm gold-coated aerogel was enhanced by LSPR over a broad wavelength range at λ > 500 nm. Besides, the enhanced absorption at λ < 500 nm is mainly caused by the interband absorption of gold (See Supplementary Fig. S6)^45^.

### SERS measurements and enhancement factor

When the light was incident on the metal surface under specific conditions, the wave may excite LSPR on the surface. This effect leads to strong amplification of the light in the near field of the surface, resulting in a large enhancement of the Raman scattering^46^.

To investigate the effect of the LSPR on the SERS signal, Raman spectra of BZT were obtained with a Raman spectrometer operating with He-Ne (633 nm) laser excitation. The excitation wavelength of 633 nm was arbitrarily chosen among the commonly used wavelengths (such as 532 nm, 633 nm and 785 nm) for Raman spectroscopy because metal-coated aerogel was expected to show SERS enhancement over the broadband wavelengths regime due to the broadband LSPR excitation.

**Figure 6:**
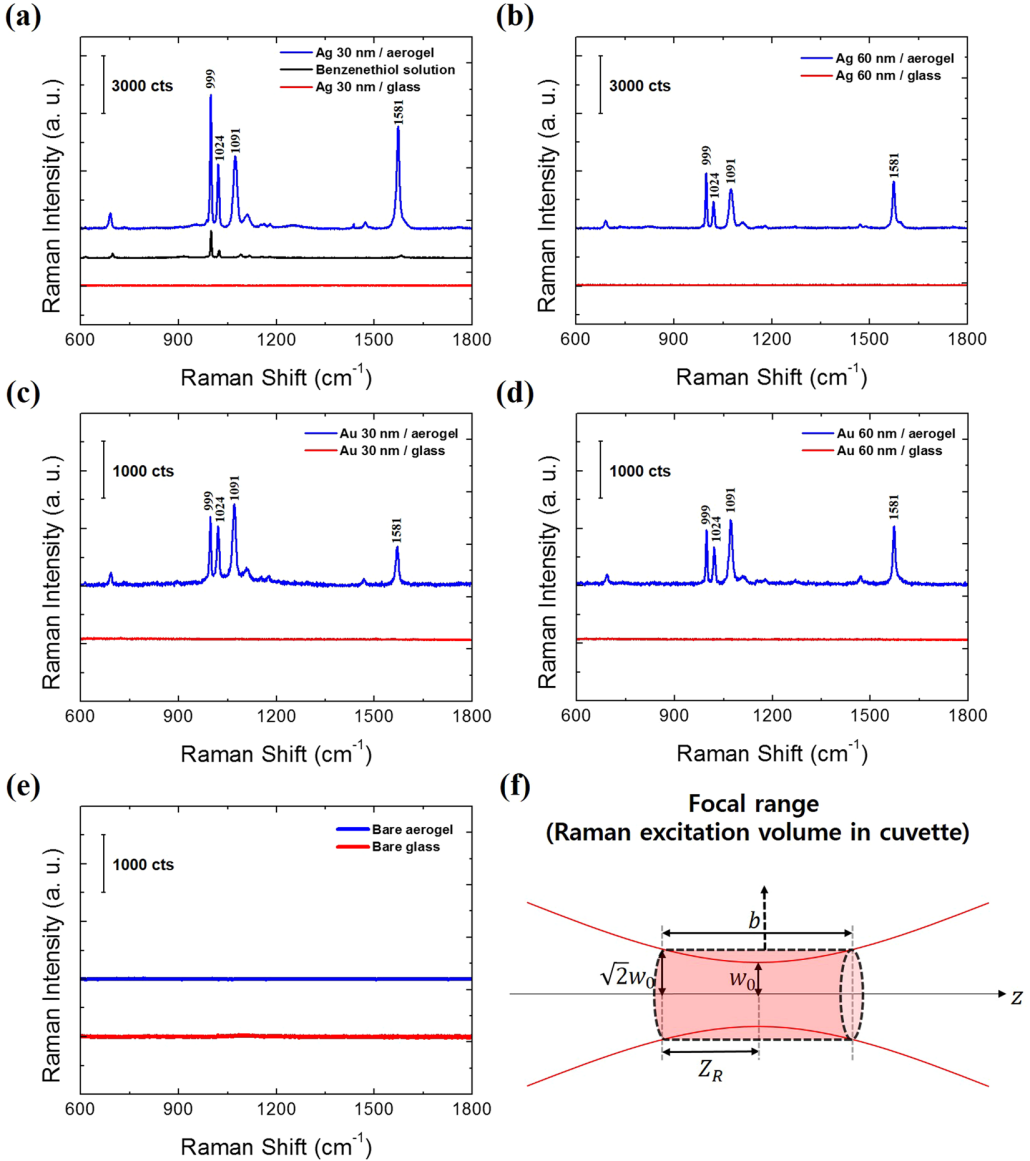
Raman spectra of benzenethiol monolayer on the metal-coated aerogel (blue) and glass (red) with different metal thickness: (**a**) Ag 30 nm, (**b**) Ag 60 nm, (**c**) Au 30 nm, (**d**) Au 60 nm. Raman spectrum of liquid benzenethiol [black in (**a**)] is given as a reference. All spectra were obtained with a 633 nm laser. (**e**) Raman spectra of benzenethiol on the bare aerogel (blue) and glass (red). (**f**) Schematic of the confocal range of the laser for calculation of the number of excited molecules in liquid benzenethiol.

As described in Fig. 6a–d, the BZT on the metal-coated aerogel exhibited strongly amplified Raman features. The enhanced Raman signals come from the strong electric field localized by metal nanogaps and electric field moved towards the sensing area caused by the ultralow index of the substrate. The BZT on the planar metal-coated glass shows indistinguishable Raman features. However, if we increase the excitation laser power into four times and the data integration time into ten times, the Raman signals from the monolayer benzenethiol on silver-coated glasses was observable, as shown in Supplementary Fig. S7. Figure 6e represents the Raman signal of benzenethiol on the bare aerogel and bare glass without the metal coating; however, the peaks were not visible. As a reference, the Raman signal from a pure (≥99%) liquid benzenethiol is also presented in Fig. 6a.

To evaluate the enhancement factors of the metal-coated aerogel substrates, we performed a normalization procedure, as shown in Fig. 6f. We assumed that Raman scattering occurred only in the focal volume of the laser beam. The total number of Raman active molecules in the liquid benzenethiol could be determined from the density (1.077 g/mL) and molar mass (110.17 g/mol) of the liquid benzenethiol in the cuvette and the volume of the focal region, approximated as a cylindrical shape, and its geometrical dimensions extracted from the Gaussian beam parameters^47^. The height of the cylinder-shaped focal region was twice the confocal parameter 2*Z*_*R*_ and the radius became the beam width at *Z*_*R*_, which was $\sqrt{2}$ times larger than the beam waist ($\sqrt{2}{{\boldsymbol{w}}}_{0}$). Therefore, the number of excited molecules could be calculated as the product of the laser excitation volume, the Avogadro number, the density of the liquid benzenethiol divided by the molar mass of the benzenethiol molecule. The number of excited benzenethiol molecules in the monolayer on the SERS substrate could be calculated in a similar way. By multiplying the area of the laser spot, the surface density of the benzenethiol monolayer and the surface factor by the geometry of the SERS substrate, we calculated the number of the excited molecules on the SERS substrate.

Through the aforementioned normalization, we calculated the enhancement factor for peaks at 999, 1,024, 1,091, and 1,580 cm^−1^, as shown in Table 1^48,49^. The enhancement factors (EF) were calculated by the following formula^38^.

$${EF}=({{I}}_{{SERS}}/{{I}}_{{lBZT}})/({{N}}_{{SERS}}/{{N}}_{{lBZT}}\,)$$

where ${{I}}_{{SERS}}$ and ${{I}}_{{lBZT}}$ are the Raman intensities of the SERS substrate and liquid benzenethiol, respectively. ${{N}}_{{SERS}}$ and ${{N}}_{{lBZT}}$ are the number of excited molecules on the SERS substrate and in the liquid benzenethiol, respectively. For the samples on the aerogel substrates coated with 30 and 60 nm Ag films, the EFs were experimentally observed as high as $7.9\times {10}^{7}\,and\,3.6\times {10}^{7}$, respectively. The larger EF and stronger SERS signal of the 30 nm Ag-coated aerogel were attributed to the higher density of hotspots per unit area compared to the 60 nm Ag-coated aerogel. 30 nm gold-coated aerogel provides experimentally measured SERS enhancement factor of $1.5\times {10}^{7}$, which is lower than silver-coated aerogels. Generally, silver nanostructure shows stronger near field enhancement than gold nanostructures because gold have larger LSPR damping due to the interband transition in the visible wavelength region^50,51^. For 30 nm Ag-coated aerogels, the experimental EF and theoretical maximum EF values from FDTD showed the same order of magnitude of 10^7^.

**Table 1:** Experimentally measured SERS enhancement factor for benzenethiol molecules adsorbed on metal-coated aerogel substrates.

Vibrational assignment	Raman shift (cm^−1^)	SERS Enhancement Factor			
		Ag 30 nm	Ag 60 nm	Au 30 nm	Au 60 nm
S-H bending + in-plane ring def.	999	9.1 × 10^6^	3.8 × 10^6^	1.6 × 10^6^	1.3 × 10^6^
in-plane ring def.	1024	1.6 × 10^7^	6.7 × 10^6^	4.9 × 10^6^	3.1 × 10^6^
C-S stretching + in-plane ring def.	1091	3.9 × 10^7^	2.2 × 10^7^	1.5 × 10^7^	1.2 × 10^7^
C-C stretching	1580	7.9 × 10^7^	3.6 × 10^7^	1.0 × 10^7^	1.5 × 10^7^

## Conclusion

We fabricated large size metal-coated aerogels with a size of a few tens of centimeters by a simple, low-cost chemical synthesis and metal deposition process. We demonstrated these highly nanoporous silica aerogels to be effective SERS templates for enhancing Raman gain, owing to their surface texture with a high density of metallic nanogaps and the ultralow refractive index of the substrate. The SERS enhancement factor reached up to $7.9\times {10}^{7}$ for BZT adsorbed on the surface of the 30 nm silver-coated aerogel.

## Electronic supplementary material

Supplementary information

## References

[1] Brian B, Sepúlveda B, Alaverdyan Y, Lechuga LM, Käll M (2009). Sensitivity enhancement of nanoplasmonic sensors in low refractive index substrates. Opt. Express.

[2] Gao Y, Gan Q, Bartoli FJ (2014). Spatially selective plasmonic sensing using metallic nanoslit arrays. IEEE J. Sel. Top. Quantum Electron..

[3] Hooshmand N, Bordley JA, El-Sayed MA (2015). Plasmonic Spectroscopy: The Electromagnetic Field Strength and its Distribution Determine the Sensitivity Factor of Face-to-Face Ag Nanocube Dimers in Solution and on a Substrate. J. Phys. Chem. C.

[4] Hooshmand N, Panikkanvalappil SR, El-Sayed MA (2016). Effects of the Substrate Refractive Index, the Exciting Light Propagation Direction, and the Relative Cube Orientation on the Plasmonic Coupling Behavior of Two Silver Nanocubes at Different Separations. J. Phys. Chem. C.

[5] Nguyen Minh-Kha, Su Wei-Nien, Hwang Bing-Joe (2017). A Plasmonic Coupling Substrate Based on Sandwich Structure of Ultrathin Silica-Coated Silver Nanocubes and Flower-Like Alumina-Coated Etched Aluminum for Sensitive Detection of Biomarkers in Urine. Advanced Healthcare Materials.

[6] Kang G, Yoo J, Ahn J, Kim K (2015). Transparent dielectric nanostructures for efficient light management in optoelectronic applications. Nano Today.

[7] Kanamori K, Aizawa M, Nakanishi K, Hanada T (2007). New transparent methylsilsesquioxane aerogels and xerogels with improved mechanical properties. Adv. Mater..

[8] Prakash SS, Brinker CJ, Hurd AJ, Rao SM (1995). Silica aerogel films prepared at ambient pressure by using surface derivatization to induce reversible drying shrinkage. Nature.

[9] Wei G, Liu Y, Zhang X, Yu F, Du X (2011). Thermal conductivities study on silica aerogel and its composite insulation materials. Int. J. Heat Mass Transf..

[10] Kanamori K, Aizawa M, Nakanishi K, Hanada T (2008). Elastic organic–inorganic hybrid aerogels and xerogels. J. Sol-Gel Sci. Technol..

[11] Hrubesh LW (1998). Aerogel applications. J. Non. Cryst. Solids.

[12] Fu T, Tang J, Chen K, Zhang F (2015). Visible, near-infrared and infrared optical properties of silica aerogels. Infrared Phys. Technol..

[13] Cheng W, Rechberger F, Niederberger M (2016). Three-Dimensional Assembly of Yttrium Oxide Nanosheets into Luminescent Aerogel Monoliths with Outstanding Adsorption Properties. ACS Nano.

[14] Karlash, A. & Ivanov, I. Optical and Photoluminecsence Properties of Powdered Silica Aerogel ar-SiO x. *Electron*. *Nanotechnol*. 105–108 (2015).

[15] Sanchez-Paradinas S (2015). Aerogels from CdSe/CdS Nanorods with Ultra-long Exciton Lifetimes and High Fluorescence Quantum Yields. Adv. Mater..

[16] Walrafen GE, Hokmabadi MS, Holmes NC, Nellis WJ, Henning S (1985). Raman spectrum and structure of silica aerogel. J. Chem. Phys..

[17] Tsujimi Y, Courtens E, Pelous J, Vacher R (1988). Raman-Scattering Measurements of Acoustic Superlocalization in Silica Aerogels. Phys. Rev. Lett..

[18] Boukenter A (1988). Vibrational modes in silica aerogels: low-frequency Raman scattering. J. Phys. C Solid State Phys..

[19] Riegel B, Hartmann I, Kiefer W, Groβ J, Fricke J (1997). Raman spectroscopy on silica aerogels. J. Non. Cryst. Solids.

[20] Anedda A (2003). Raman Investigation of Surface OH-Species in Porous Silica. J. Phys. Chem. B.

[21] Burchell MJ (2006). Identification of minerals and meteoritic materials via Raman techniques after capture in hypervelocity impacts on aerogel. J. Raman Spectrosc..

[22] Racheli, R., David, G., Katya, R. & Adi, S. Direct Fabrication of 3D Metallic Networks and Their Performance. *Adv*. *Mater*. **29**, (2017). 10.1002/adma.201604018 27943494

[23] Landau, L. D., Lifshitz, E. M. & Pitaevskii, L. P. *Electrodynamics of continuous media*. (Pergamon Press, 1984).

[24] Jackson, J. D. *Classical Electrodynamics*. (John Wiley & Sons, Ltd., 1999).

[25] Biggs KB, Camden JP, Anker JN, Duyne RP (2009). Van. Surface-Enhanced Raman Spectroscopy of Benzenethiol Adsorbed from the Gas Phase onto Silver Film over Nanosphere Surfaces: Determination of the Sticking Probability and Detection Limit Time. J. Phys. Chem. A.

[26] Sun F, Galvan DD, Jain P, Yu Q (2017). Multi-functional, thiophenol-based surface chemistry for surface-enhanced Raman spectroscopy. Chem. Commun..

[27] Poirier GE, Pylant ED (1996). The Self-Assembly Mechanism of Alkanethiols on Au(111). Science (80-.)..

[28] Vericat C, Vela ME, Benitez G, Carro P, Salvarezza RC (2010). Self-assembled monolayers of thiols and dithiols on gold: new challenges for a well-known system. Chem. Soc. Rev..

[29] Salazar Alarcón L (2013). Growth of 1,4-Benzenedimethanethiol Films on Au, Ag, and Cu: Effect of Surface Temperature on the Adsorption Kinetics and on the Single versus Multilayer Formation. J. Phys. Chem. C.

[30] Talley CE, Jusinski L, Hollars CW, Lane SM, Huser T (2004). Intracellular pH Sensors Based on Surface-Enhanced Raman Scattering. Anal. Chem..

[31] Sun F (2014). Sensitive and Fast Detection of Fructose in Complex Media via Symmetry Breaking and Signal Amplification Using Surface-Enhanced Raman Spectroscopy. Anal. Chem..

[32] Linn NC, Sun C-H, Arya A, Jiang P, Jiang B (2009). Surface-enhanced Raman scattering on periodic metal nanotips with tunable sharpness. Nanotechnology.

[33] Shin D (2017). Scalable variable-index elasto-optic metamaterials for macroscopic optical components and devices. Nat. Commun..

[34] Gupta G, Kondoh J (2007). Tuning and sensitivity enhancement of surface plasmon resonance sensor. Sensors Actuators B Chem..

[35] Solís DM, Taboada JM, Obelleiro F, Liz-Marzán LM, García de Abajo FJ (2014). Toward Ultimate Nanoplasmonics Modeling. ACS Nano.

[36] Le R, E. C. & Etchegoin, P. G. *Principles of Surface-Enhanced Raman Spectroscopy*. (Elsevier). 10.1016/B978-0-444-52779-0.X0001-3 (2009).

[37] Le REC, Etchegoin PG (2006). Rigorous justification of the |E|4 enhancement factor in Surface Enhanced Raman Spectroscopy. Chem. Phys. Lett..

[38] Le REC, Blackie EJ, Meyer M, Etchegoin PG (2007). Surface Enhanced Raman Scattering Enhancement Factors: A Comprehensive Study. J. Phys. Chem. C.

[39] Schlücker S (2014). Surface-Enhanced Raman Spectroscopy: Concepts and Chemical Applications. Angew. Chemie Int. Ed..

[40] Roelli P, Galland C, Piro N, Kippenberg TJ (2016). Molecular cavity optomechanics as a theory of plasmon-enhanced Raman scattering. Nat Nano.

[41] Schmidt MK, Esteban R, Gonzalez-Tudela A, Giedke G, Aizpurua J (2016). Quantum Mechanical Description of Raman Scattering from Molecules in Plasmonic Cavities. ACS Nano.

[42] Bosman M, Anstis GR, Keast VJ, Clarke JD, Cortie MB (2012). Light Splitting in Nanoporous Gold and Silver. ACS Nano.

[43] Shao B (2017). Upconversion emission enhancement by porous silver films with ultra-broad plasmon absorption. Opt. Mater. Express.

[44] Ryu Y, Kang G, Lee C-W, Kim K (2015). Porous metallic nanocone arrays for high-density SERS hot spots via solvent-assisted nanoimprint lithography of block copolymer. RSC Adv..

[45] Li XD, Chen TP, Liu Y, Leong KC (2014). Influence of localized surface plasmon resonance and free electrons on the optical properties of ultrathin Au films: a study of the aggregation effect. Opt. Express.

[46] Stiles PL, Dieringer JA, Shah NC, Van Duyne RP (2008). Surface-Enhanced Raman Spectroscopy. Annu. Rev. Anal. Chem..

[47] Chrimes AF, Khoshmanesh K, Stoddart PR, Mitchell A, Kalantar-zadeh K (2013). Microfluidics and Raman microscopy: current applications and future challenges. Chem. Soc. Rev..

[48] Tai HJ, Myung Soo K, Kwan K (1987). Surface-enhanced Raman scattering of benzenethiol in silver sol. J. Raman Spectrosc..

[49] Scott DW (1956). Benzenethiol: Thermodynamic Properties in the Solid, Liquid and Vapor States; Internal Rotation of the Thiol Group1. J. Am. Chem. Soc..

[50] Suzuki S, Yoshimura M (2017). Chemical Stability of Graphene Coated Silver Substrates for Surface-Enhanced Raman Scattering. Sci. Rep..

[51] Khaywah MY (2015). Ultrastable, Uniform, Reproducible, and Highly Sensitive Bimetallic Nanoparticles as Reliable Large Scale SERS Substrates. J. Phys. Chem. C.

