# Supplementary material for: Large-scale nanoporous metal-coated silica aerogels for high SERS effect improvement
Source: Sci Rep. 2018 Oct 11;8:15144. doi: 10.1038/s41598-018-33539-z (PMC6181977; doi:10.1038/s41598-018-33539-z)
Supplement: Supplementary file 1 — Supplementary information [file 41598_2018_33539_MOESM1_ESM.docx]

Supplementary Information

Large-scale nanoporous metal-coated silica aerogels for high SERS effect improvement

Changwook Kim^‡a^, Seunghwa Baek^‡a^, Yunha Ryu^a^, Yeonhong Kim^a^, Dongheok Shin^a^, Changwon Lee^b^, Wounjhang Park^c^, Augustine M. Urbas^d^, Gumin Kang^*e^ and Kyoungsik Kim^*a^

^a^ School of Mechanical Engineering, Yonsei University, 50 Yonsei-ro, Seodaemun-gu, Seoul, 03722, Republic of Korea.

^b^ School of Basic Sciences, Hanbat National University, Daejeon, Republic of Korea.

^c^ Department of Electrical, Computer & Energy Engineering, University of Colorado, Boulder, CO 80309, USA

^d^ Materials and Manufacturing Directorate, Air Force Research Laboratory, Wright-Patterson AFB, OH 45433, USA

^e^ Nanophotonics Research Center, Korea Institute of Science and Technology (KIST), Seoul 02792, Republic of Korea.

* email: [kks@yonsei.ac.kr](mailto:kks@yonsei.ac.kr), guminkang@kist.re.kr

^‡^ These authors contributed equally to this work.


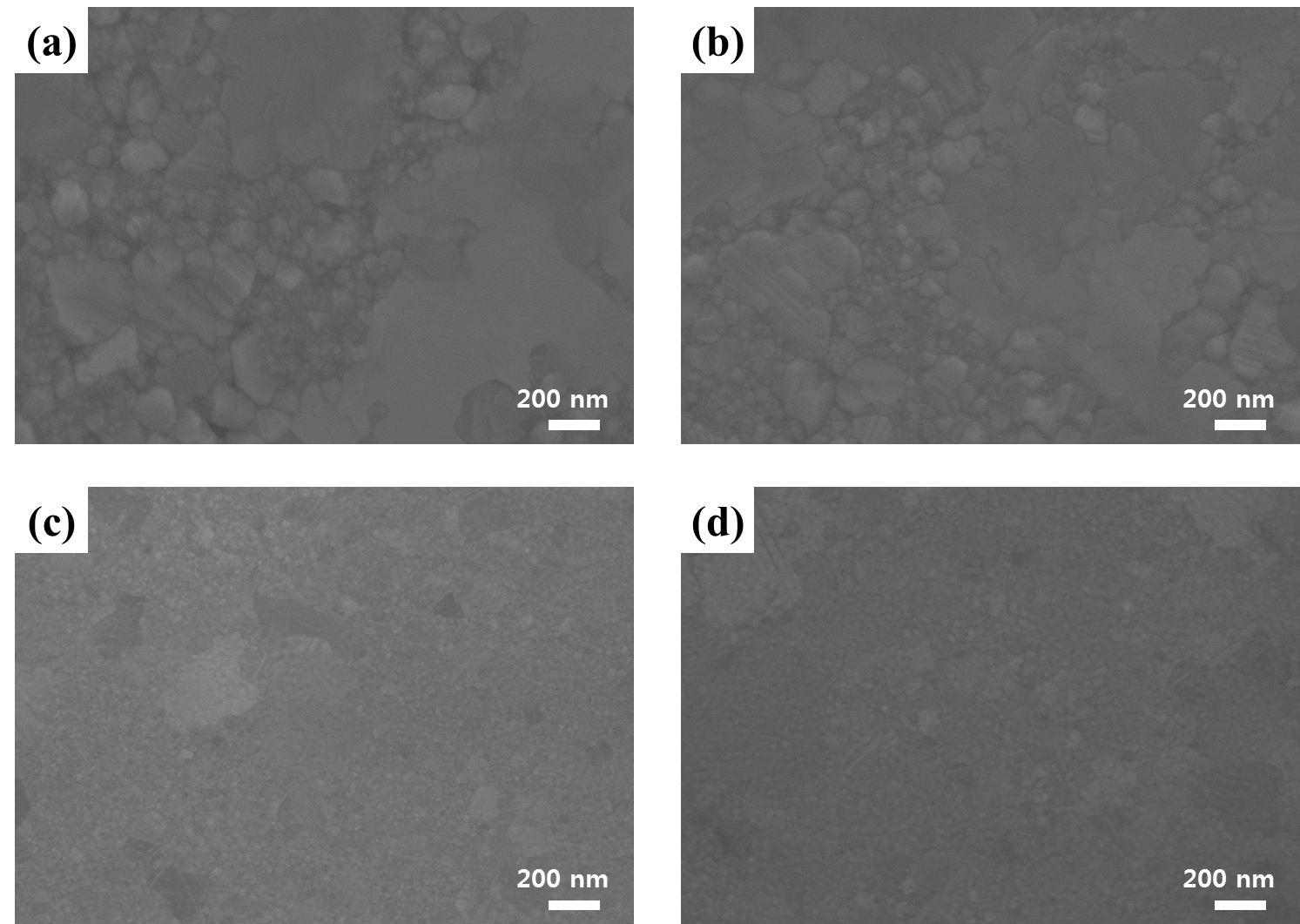


**Figure S1.** SEM images of silver-coated glass surface with different Ag thickness; (a) 30, (b) 60 nm. SEM images of gold-coated glass surface with different Au thickness; (g) 30, (h) 60 nm.


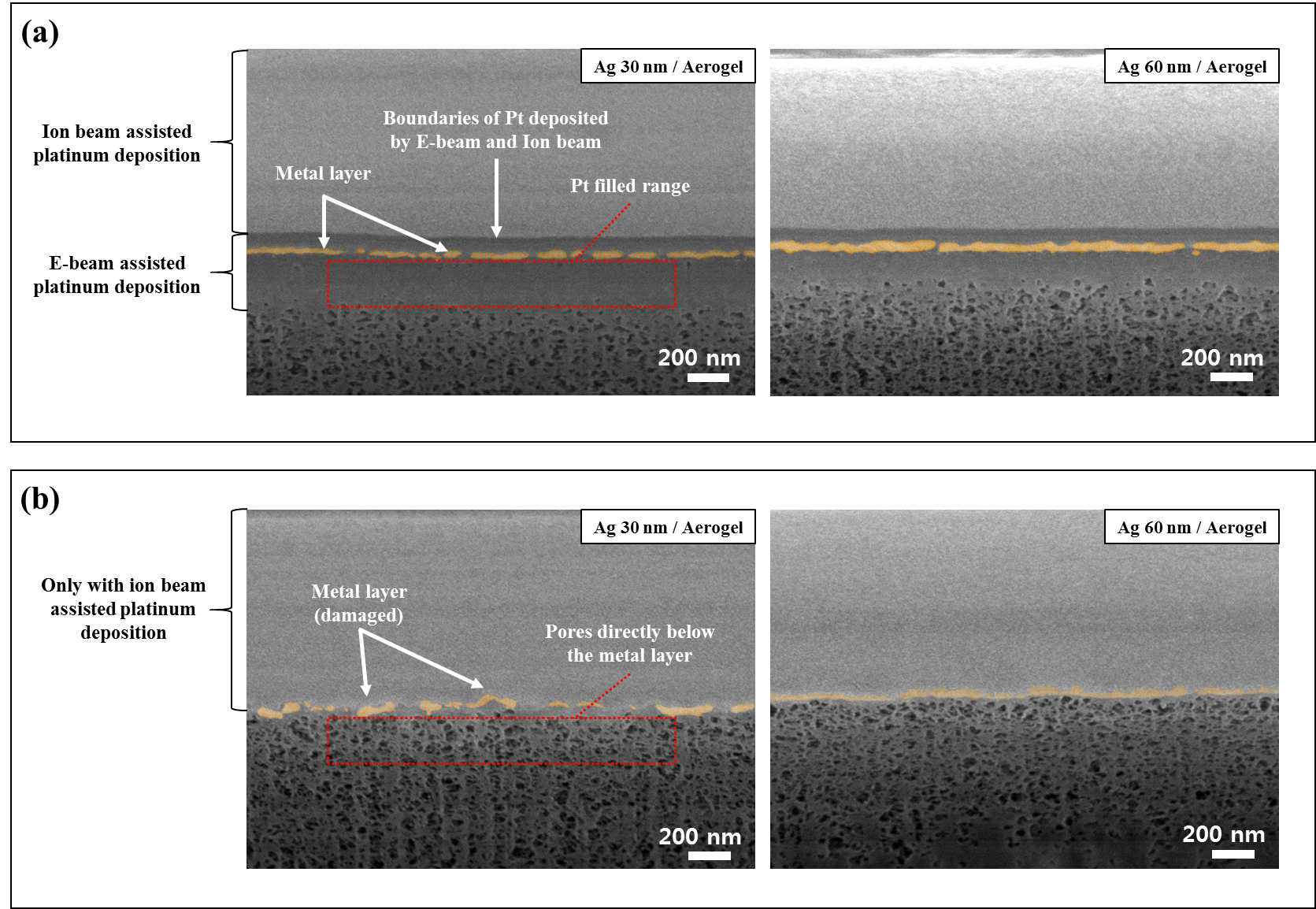


**Figure S2.** (a) Cross sectional images of the Silver-coated aerogels using both electron beam and ion beam assisted platinum deposition before FIB milling. (b) Cross sectional images of the Silver-coated aerogels using ion beam assisted platinum deposition only before FIB milling. In the FIB milling process, Platinum is deposited to protect the surface by two different methods: Electron beam and Ion beam assisted Pt deposition. E-beam assisted Pt deposition has low deposition rate, but does not damage the surface and the deposition becomes finer. Therefore, if the structure is porous, such as metal-coated aerogels, the Pt atoms penetrate into the surface and pores of aerogel located directly below metal thin film are filled with Pt. On the other hand, when Pt is deposited by ion beam method which has high deposition rate, pores are not filled with Pt. It can be seen that the pores of aerogel are located directly below the metal film. However ion beam assisted Pt deposition can damage the surface.^S1^


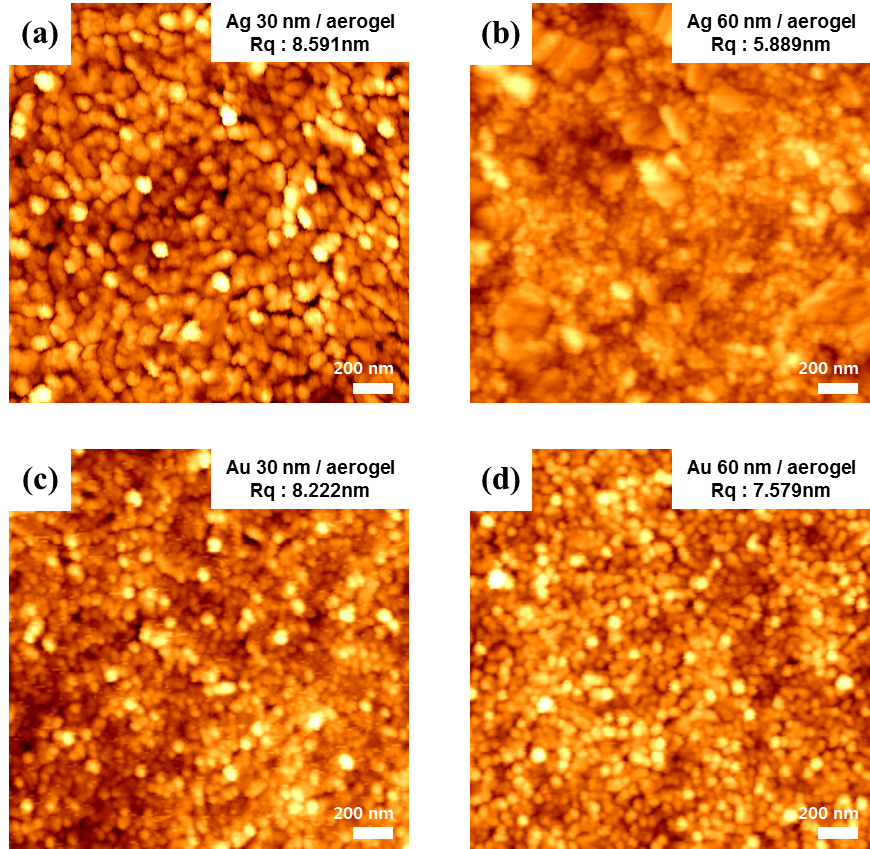


**Figure S3.** AFM images of the (a) 30-nm, (b) 60-nm silver-coated aerogel surface and (c) 30-nm, (d) 60-nm gold-coated aerogel surface. Evaluated root mean squared roughness (Rq) were indicated in the inset.


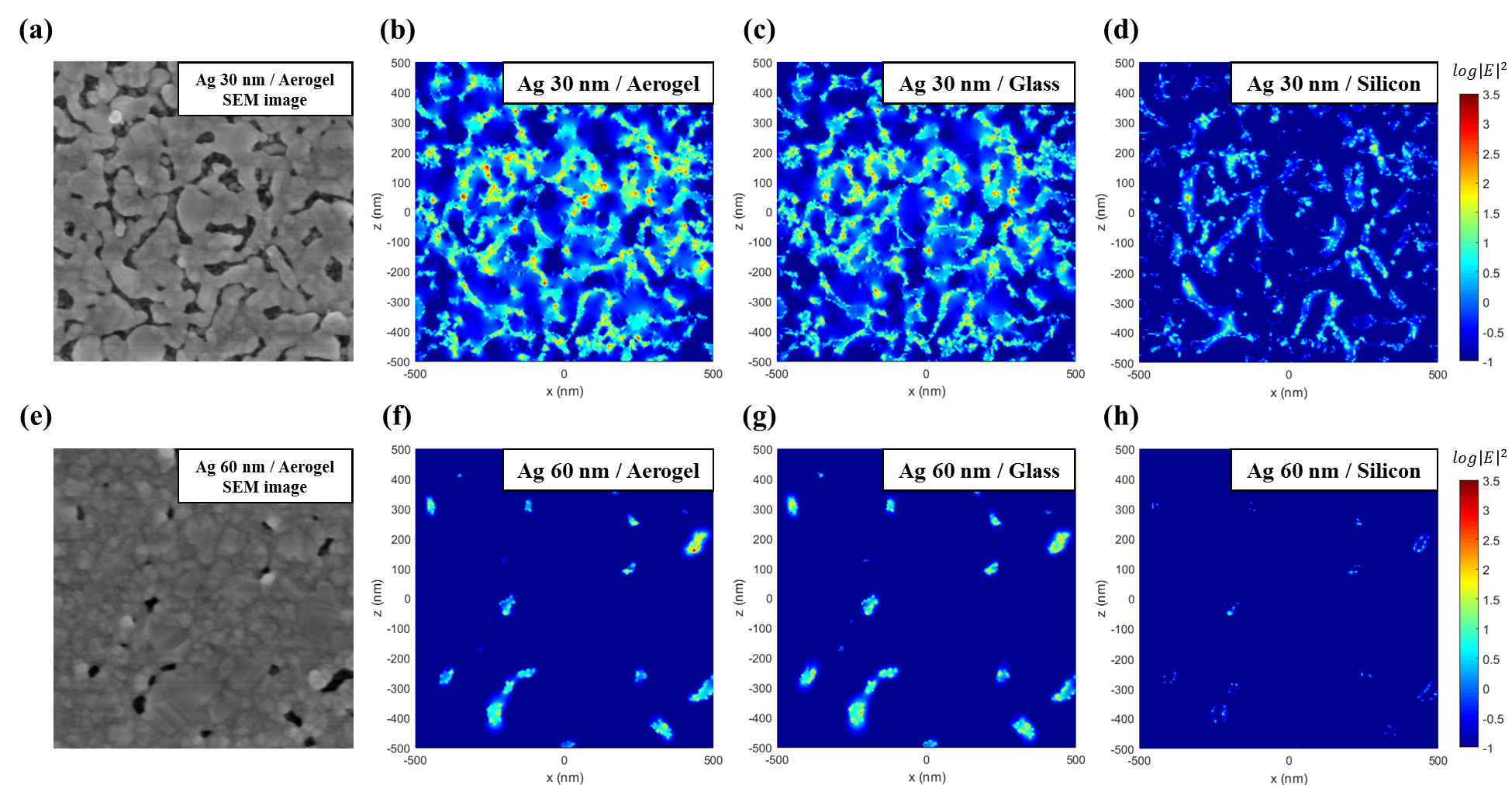


**Figure S4.** (a) SEM image of the 30-nm silver-coated aerogel surface for FDTD simulation. This SEM image was imported into the FDTD simulation.^S2^ (b)~(d) Electric field profiles by 532-nm light excitation on the top surface of 30 nm silver-coated (b) Ag/Aerogel, (c) Ag/glass, and (d) Ag/Si. (e) SEM image of the 60-nm silver-coated aerogel surface for FDTD simulation. (f)~(h) Electric field profiles by 532-nm light excitation on the top surface of the 60-nm silver-coated (f) Ag/Aerogel, (g) Ag/glass, and (h) Ag/Si.

**
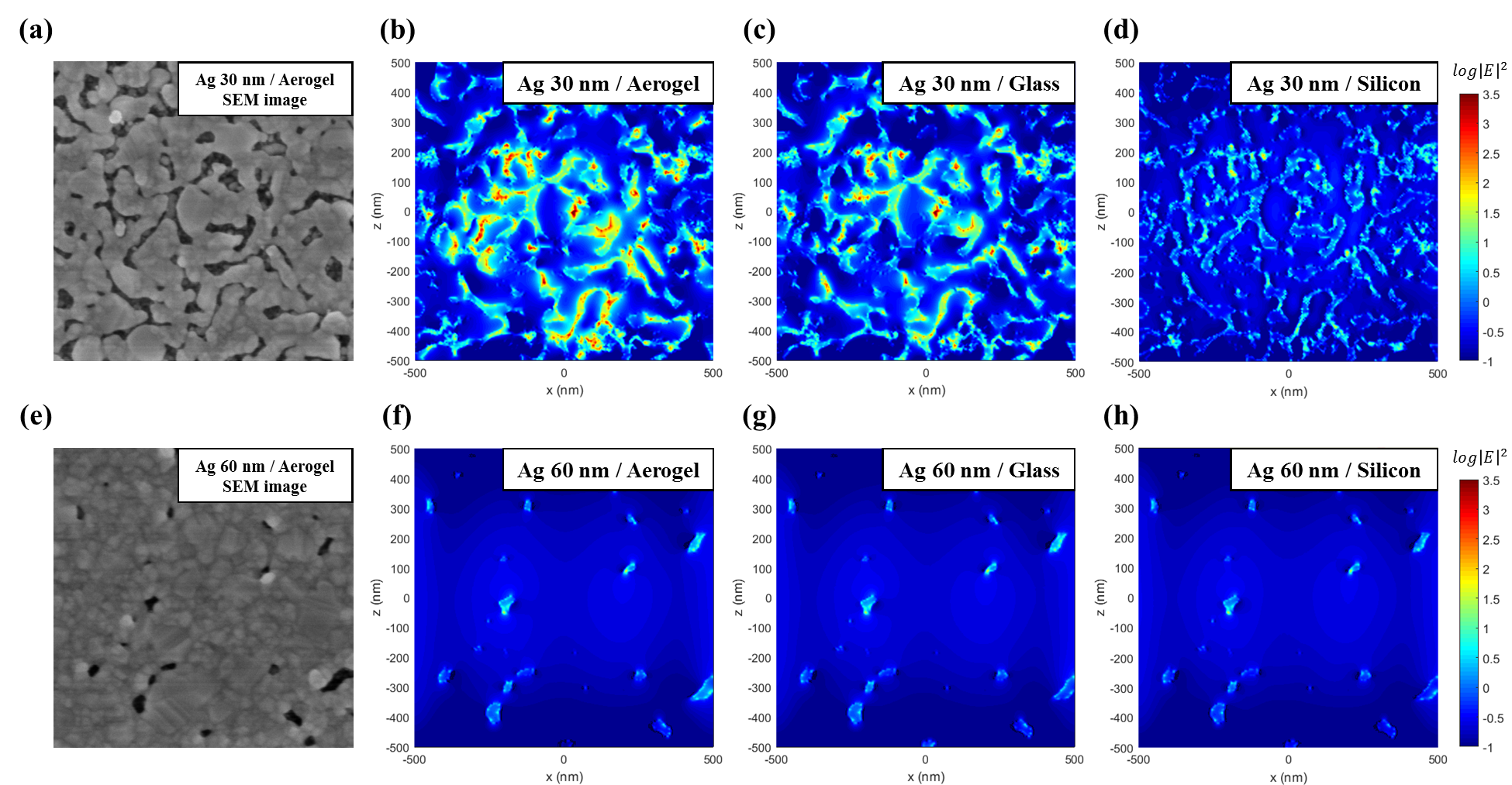
**

**Figure S5.** (a) SEM image of the 30-nm silver-coated aerogel surface for FDTD simulation. This SEM image was imported into the FDTD simulation.^S2^ (b)~(d) Electric field profiles by 785-nm light excitation on the top surface of 30 nm silver-coated (b) Ag/Aerogel, (c) Ag/glass, and (d) Ag/Si. (e) SEM image of the 60-nm silver-coated aerogel surface for FDTD simulation. (f)~(h) Electric field profiles by 785-nm light excitation on the top surface of the 60-nm silver-coated (f) Ag/Aerogel, (g) Ag/glass, and (h) Ag/Si.


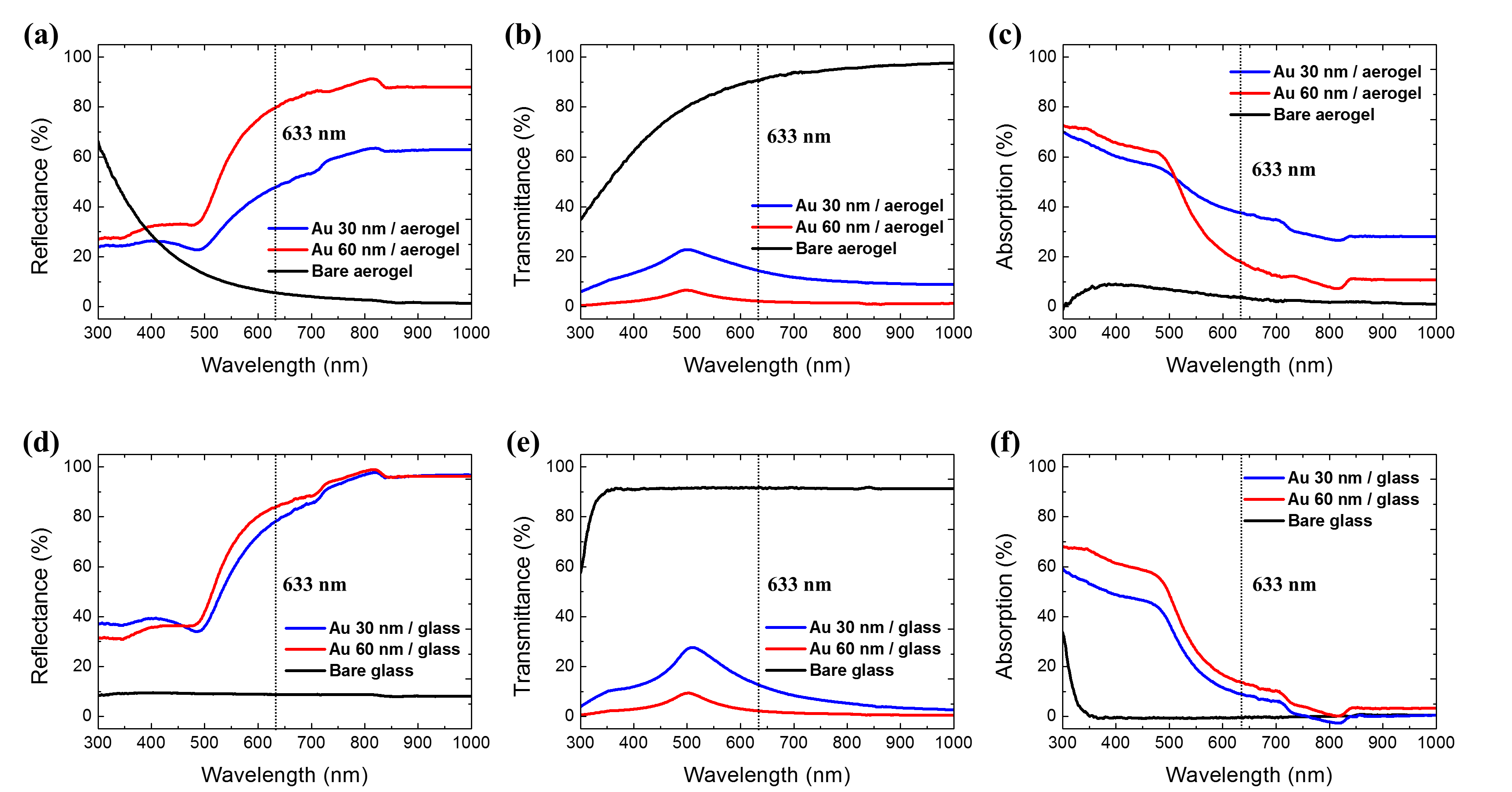


**Figure S6.** Measured optical (a) reflectance, and (b) transmittance spectra of the bare aerogel (black), 30-nm gold-coated aerogel (red), and 60 nm gold-coated aerogel (blue). (c) Absorption spectra of the bare (black), 30 nm (blue) and 60 nm (red) gold-coated aerogel calculated from 1-R-T. Measured optical (d) reflectance, and (e) transmittance spectra of the bare glass (black), 30-nm gold-coated glass (red), and 60-nm gold-coated glass (blue). (f) Absorption spectra of the bare (black), 30-nm (blue) and 60-nm (red) gold-coated glass calculated from 1-R-T. Black dotted line indicates excitation wavelength (633 nm).


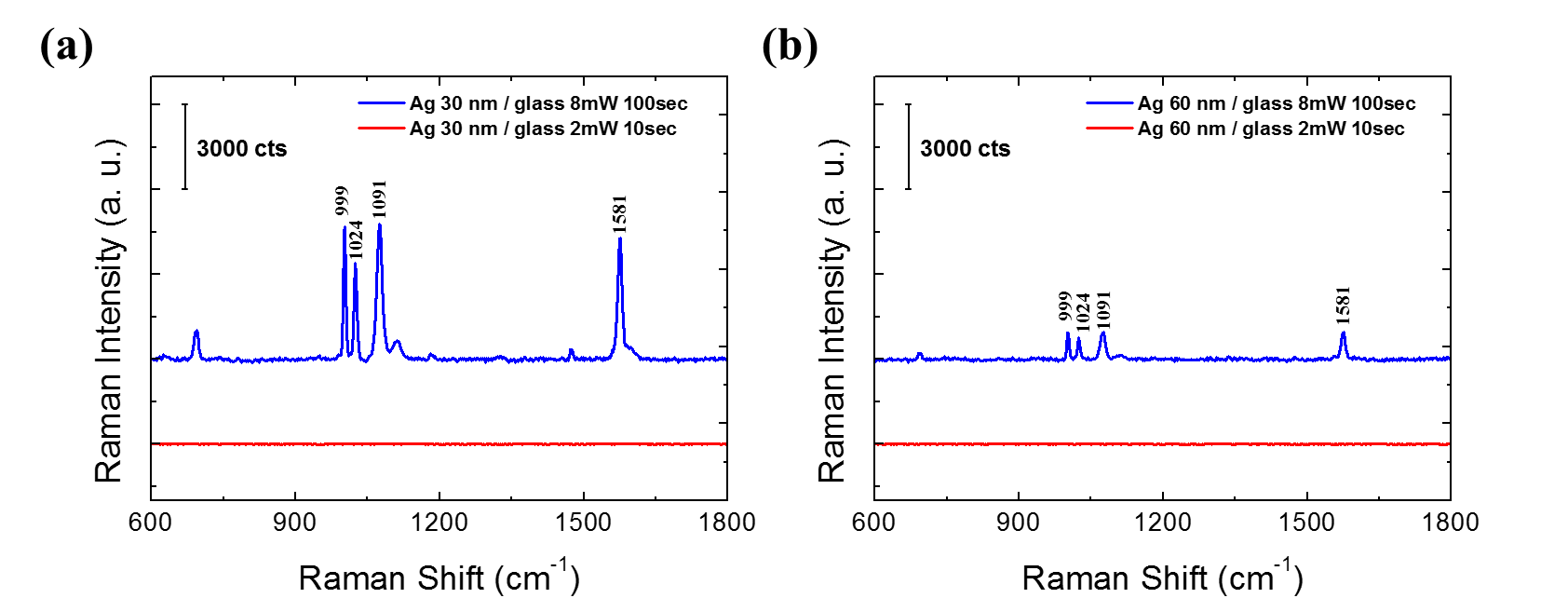


**Figure S7.** Measured Raman spectra of the benzenethiol monolayer on the silver deposited glass with different thickness: (a) 30 nm, (b) 60 nm by increasing the laser power to 4 times and the integration time to 10 times (blue) as compared with the original measurement (red). All spectra were obtained with a 633-nm laser.

REFERENCES

S1. Kwong, W. Y. & Zhang, W. Y. Electron-beam assisted platinum deposition as a protective layer for FIB and TEM applications. in ISSM 2005, IEEE International Symposium on Semiconductor Manufacturing, 2005. 469–471 (2005). doi:10.1109/ISSM.2005.1513408

S2. Solís, D. M.; Taboada, J. M.; Obelleiro, F.; Liz-Marzán, L. M.; García de Abajo, F. J. Toward Ultimate Nanoplasmonics Modeling. *ACS Nano* **2014**, *8*, 7559–7570.
